# Transcriptomic Analysis of *Trachinotus ovatus* Under Flow Velocity Stress

**DOI:** 10.3390/ani15131932

**Published:** 2025-06-30

**Authors:** Jing Zhang, Xixi Liu, Jiayue Dai, Sufang Niu, Xuefeng Wang, Baogui Tang

**Affiliations:** 1 College of Fisheries, Guangdong Ocean University, Zhanjiang 524088, China; zjouzj@126.com (J.Z.); ayliuxixi@163.com (X.L.); daijiayue1998@163.com (J.D.); wolf0487@126.com (S.N.); xuefeng1999@126.com (X.W.); 2 Guangdong Provincial Key Laboratory of Aquatic Animal Disease Contril and Healthy Culture, College of Fishery, Guangdong Ocean University, Zhanjiang 524088, China

**Keywords:** *Trachinotus ovatus*, flow velocity stress, transcriptomic analysis

## Abstract

*Trachinotus ovatus* is a euryhaline, warm-water pelagic fish species with strong adaptability, rapid growth, and a high survival rate, making it one of the most important marine aquaculture species in China. In recent years, extensive experience has been accumulated in the cage farming of *T*. *ovatus*, but whether it can adapt to deep-sea environments and grow normally remains a current research focus. This study used RNA-Seq sequencing technology to analyze the gene expression changes in the liver of *T*. *ovatus* under three conditions: rest (0 cm/s), medium flow velocity (54 cm/s), and high flow velocity (90 cm/s). Through differential expression analysis, Short Time-series Expression Miner (STEM) analysis and protein–protein interaction (PPI) network analysis, a total of 5107 differentially expressed genes (DEGs), three significantly expressed gene profiles (profile6, profile1, and profile5), and 15 hub genes were identified. The results showed that changes in flow speed significantly impacted key biological processes such as energy metabolism, protein homeostasis, and endoplasmic reticulum (ER) stress response. Under moderate and high flow conditions, glycolysis-related genes were upregulated to meet the energy demands of swimming, while the downregulation of the *PPARγ*-*RXRG* complex and its downstream genes in the lipid metabolism pathway suggested a limitation in its fatty acid β-oxidation capacity. At the same time, protein synthesis was enhanced, and the unfolded protein response (UPR) was activated to help cope with ER stress. Furthermore, when the flow speed reached 90 cm/s, the expression of UPR- related genes and the anti-apoptotic factor *JNK* significantly decreased, suggesting that the stress response was nearing its limit and could potentially trigger cell apoptosis. These findings provide new insights into the molecular adaptation mechanisms of *T*. *ovatus* to flow speed stress and offer theoretical support for its rational farming in deep-sea cages, suggesting that the water flow speed in farming should not exceed 90 cm/s.

## Simple Summary

Water flow is an important environmental factor affecting fish physiology. In this study, we performed liver transcriptome sequencing of *Trachinotus ovatus* to explore their molecular responses under different flow velocity stresses. The results revealed that moderate to high flow velocities significantly affected key biological processes, including energy metabolism, protein homeostasis, and endoplasmic reticulum stress. Notably, a high flow velocity of 90 cm/s appeared to serve as a critical threshold inducing these physiological responses. In practical applications, deep and open-sea aquaculture often faces challenges such as strong currents and complex hydrodynamic conditions. Therefore, our findings provide new insights into the molecular adaptation mechanisms of *T*. *ovatus* under flow velocity stress, and offer a valuable reference for the site selection of deep-sea cage aquaculture systems.

## 1. Introduction

Flow velocity is a critical factor affecting production and management in fish farming, with significant impacts on growth, behavior, health, and physiology [1]. Flow velocity influences fish growth and energy metabolism, as moderate speeds encourage continuous, steady swimming that promotes muscle development [2,3,4]. This activity strengthens the aerobic capacity of white muscle while reducing fat deposition [5,6]. Studies have shown that species like large yellow croaker (*Larimichthys crocea*) exhibit better growth rates at moderate flow speeds (e.g., 30–50 cm/s), while higher speeds (e.g., above 70 cm/s) increase metabolic demands, potentially hindering growth due to stress and energy depletion [7,8].

Increased flow velocities can elevate stress in fish, as indicated by higher cortisol levels, which compromise immune function, increase susceptibility to disease, and potentially raise mortality rates [9]. For example, research on rainbow trout (*Oncorhynchus mykiss*) demonstrated that high flow conditions elevated cortisol and stress responses, weakening immunity [10]. Additionally, high flow rates reduce mucus secretion on the skin, especially around the gills, making fish more vulnerable to infections [6,11].

Moderate flow conditions encourage healthy swimming behavior, which enhances swimming ability and conserves energy [12,13]. For instance, golden pompano (*T*. *ovatus*) displayed consistent, low-energy swimming under moderate flow conditions, promoting aerobic conditioning and efficient energy use [14]. Conversely, low flow rates can reduce swimming capacity, while high flow rates may cause excessive energy expenditure, disrupt feeding behavior, and reduce the feed conversion efficiency [15,16]. Increased flow velocity also improves dissolved oxygen levels in the water, facilitating oxygen uptake to support metabolic activity. However, very high flow velocities can increase oxygen consumption rapidly, leading to fatigue and reduced growth [17].

In summary, flow velocity affects fish in a “U-shaped” manner: moderate flow speeds optimize growth, health, and feed efficiency, while both low and high flow velocities can have adverse effects. Consequently, managing flow velocities in aquaculture systems is crucial for promoting optimal fish growth and health.

*T*. *ovatus* is a migratory fish widely distributed along the South China Sea coastline [18]. It has become a vital cultured marine species in the Asia–Pacific region due to its fast growth, adaptability, high survival rate, short breeding cycle, and quality meat [19,20,21]. This species thrives in cage and pond cultures, as matching feed can be used throughout the production process. Its cultivation history dates back to the 1990s, with offshore cage cultures along China’s southern coast expanding significantly [22,23]. To support the sustainable development of China’s marine aquaculture industry and reduce near-shore pollution, the government has issued directives promoting deep-sea aquaculture, where *T. ovatus* is a key species [14].

Currently, the deep-water cage culture of *T*. *ovatus* generally occurs in near-shore waters up to 20 m deep, often in bays or areas sheltered by islands and reefs, providing more stable environmental conditions [24,25]. However, expanding into deeper, offshore waters presents challenges, as conditions such as the flow velocity, wind, waves, and lack of shelter could greatly impact the cultured fish and equipment. Although offshore cage and factory culture techniques have matured, the adaptability of fish to deep-sea environments remains a primary concern. Therefore, understanding fish swimming abilities and environmental adaptability is essential for selecting species suitable for deep-sea aquaculture.

Research into the impact of flow velocity on fish commonly involves behavioral observations, metabolic rate measurements, and the assessment of physiological and biochemical indicators. With the emergence of transcriptomic technologies, researchers increasingly employ these methods to explore underlying mechanisms. However, specific molecular mechanisms related to flow velocity stress are still under investigation.

In its natural habitat, *T*. *ovatus* is widely distributed in the coastal and offshore waters of the South China Sea, where the water flow velocity is influenced by tides, ocean currents, and seasonal variations. Although direct data on flow velocities in its wild habitats are currently limited, previous studies have reported that *T. ovatus* exhibits favorable growth performance and adaptability in deep-sea cage culture systems with flow velocities ranging from 50 to 65 cm/s [26,27].

Given our limited understanding of the effects of flow velocity on *T. ovatus*, researchers have examined how flow velocity impacts its swimming behavior and exercise physiology [14]. Building upon our previous research on the effects of flow velocity on the swimming behavior and exercise physiology of *T*. *ovatus* [14], the present study further employed comparative transcriptomic analysis to investigate the dynamic changes in differentially expressed genes (DEGs) in liver tissue under varying flow velocity conditions. The aim was to elucidate the molecular adaptation mechanisms by which fish respond to flow velocity stress.

The liver plays a central role in metabolism regulation, energy homeostasis, and the stress response in fish, making it a key target organ for environmental stress studies [28,29]. As the primary site for nutrient transformation and detoxification, the liver is highly sensitive to external environmental changes. Therefore, selecting the liver for transcriptomic analysis provides a comprehensive view of the systemic physiological adaptations of fish to different flow velocity stressors.

## 2. Materials and Methods

### 2.1. Ethics Statement

This study was conducted in strict accordance with the “Guidelines for ethical review of experimental animal welfare” and the “Guidelines for Experimental Animals” (Approval number: 0501-2021).

### 2.2. Velocity Stress Experiment and Sample Collection

A total of 100 healthy juvenile *T*. *ovatus* were provided by Zhanjiang Hist Aquaculture Technology Co. Ltd. (Zhanjiang, China), with an average body length of (11.06 ± 0.70) cm and body weight of (56.09 ± 9.99) g. To reduce the stress caused by transportation and handling, fish were pre-acclimated in 1000 L tanks for 7 days before the experiment. During the acclimation period, the water temperature was maintained at (27.00 ± 1.50) °C, dissolved oxygen was kept above 8.0 mg/L, and the salinity was (30.00 ± 1.50). All fish were fasted for 24 h prior to swimming trials.

The experiment was conducted using a large swim tunnel (Swim tunnel respirometer, Loligo Systems, Viborg, Denmark) with a test section measuring 40 cm × 10 cm × 10 cm and an effective volume of approximately 10 L. The flow field in the test chamber was uniform and stable, with flow velocities ranging from 5 to 120 cm/s. It is equipped with a Witrox terminal (Loligo Systems, Viborg, Denmark) for temperature and dissolved oxygen monitoring, a DAQ-BT Bluetooth data acquisition terminal, and AutoResp™ version 2.3.0 software for system control and data recording.

The flow velocity was adjusted by controlling the rotation speed of the propeller, and water flow was passed through a honeycomb flow straightener to ensure laminar flow conditions. The flow rates were calibrated before experiments and continuously monitored during trials to ensure accuracy and stability. A Sony video camera (FDR-AX53, Sony Corporation, Tokyo, Japan) was mounted above the tunnel to record fish swimming behavior and stress duration in real time, ensuring data accuracy and reliability.

A preliminary experiment was conducted to assess the swimming capacity of *T*. ovatus using the stepwise increasing flow velocity method. Individual fish were placed in the swim tunnel, facing the water flow, and allowed to acclimate for 1 h. The flow velocity was then increased by 1 cm/s every 5 s, and swimming behavior was observed. The induced swimming speed (*U*_induced_), critical swimming speed (*U*_criti_), and burst swimming speed (*U*_burst)_ were recorded. Among these, the critical swimming speed serves as a key indicator of aerobic swimming performance. The average *U*_criti_ measured in this experiment was (99.78 ± 12.66) cm/s [14].

Based on the swimming capacity results of *T*. ovatus, three representative flow velocities were selected for the stress experiment: the static water group (LC, 0 cm/s), simulating traditional aquaculture conditions and serving as the control; the medium velocity group (LM, 60% *U*_criti_, 54 cm/s), representing a high hydrodynamic environment within the tolerance range; and the high velocity group (LH, 100% *U*_criti_, 90 cm/s), simulating strong flow stress conditions near the upper limit of sustained swimming ability.

Each group consisted of six fish tested individually. Prior to the trial, each fish was acclimated in static water for 1 h, then exposed to the target velocity for 20 min to induce flow stress [30]. After the stress period, fish were immediately transferred into a bucket containing 100 mg/L eugenol solution for anesthesia. Liver tissues were rapidly collected and frozen in liquid nitrogen for transcriptomic analysis. Liver tissues from every two fish were pooled prior to RNA extraction, yielding three biological replicates per group (6 fish per group, 3 pooled samples in total).

### 2.3. RNA Extraction, Library Construction, and Sequencingz

To obtain sufficient RNA quantities and minimize individual variability, a pooling strategy was employed. Specifically, six liver samples from each group (three groups in total) were equally mixed in pairs, resulting in three biological pooled samples per group (3 groups × 3 samples = 9 pooled samples). Total RNA was extracted from these nine pooled samples using TRIzol reagent (Invitrogen, Carlsbad, CA, USA) following the manufacturer’s standard protocol. The concentration and purity of the total RNA were assessed using RNase-free agarose gel electrophoresis, a NanoDrop 2000 spectrophotometer(Thermo Fisher Scientific, Wilmington, DE, USA), and an Agilent 2100 Bioanalyzer (Agilent Technologies, Palo Alto, CA, USA) to ensure RNA integrity and the absence of contamination.

Polyadenylated mRNA was enriched from total RNA using oligo(dT)-attached magnetic beads, and then fragmented into short fragments using ultrasonic shearing. Using the fragmented mRNA as a template, first-strand cDNA was synthesized with random hexamer primers and M-MuLV reverse transcriptase. Subsequently, the RNA strand was degraded using RNase H and the second-strand cDNA was synthesized using DNA polymerase I with dNTPs. The resulting double-stranded cDNA was purified, end-repaired, A-tailed, and ligated with sequencing adapters. Fragments approximately 200 bp in length were selected using Hieff NGS® DNA Selection Beads (Yeasen Biotechnology, Shanghai, China), followed by PCR amplification and another round of purification with Hieff NGS® DNA Selection Beads to obtain the final cDNA library. High-throughput sequencing of the cDNA libraries was conducted on the Illumina sequencing platform by Genedenovo Biotechnology Co., Ltd. (Guangzhou, China).

### 2.4. Quality Control and Read Alignment

Raw sequencing data were subjected to a stringent quality control (QC) pipeline. Initially, the software fastp version 0.18.0 [31] was used to filter the raw reads. The filtering process included the removal of reads containing adapter sequences, the elimination of reads with more than 10% unknown nucleotides (N), the exclusion of reads composed entirely of adenine (poly-A reads), and the removal of low-quality reads (where over 50% of bases had a Phred quality score ≤ 20). Clean reads were evaluated based on Q30 values and the GC content to ensure sequencing quality. Subsequently, the clean reads were aligned to the *T. ovatus* ribosomal RNA database using Bowtie2 [32], and only unmapped reads were retained for downstream analysis.

The genome assembly and gene annotation files of *T. ovatus* were downloaded from Figshare [33] and used to construct the index of the reference genome. High-quality clean reads were then aligned to the *T*. *ovatus* reference genome using HISAT2 [34]. Based on the reference genome annotation, the gene expression levels were quantified, and functional annotation analyses were performed to support downstream transcriptomic interpretation.

### 2.5. Identification and Selection of Differentially Expressed Genes (DEGs)

The transcript expression levels for each sample were normalized using the FPKM (Fragments Per Kilobase of transcript per Million mapped reads) algorithm. Principal Component Analysis (PCA) with a 95% confidence level was conducted to assess the similarity in expression profiles among the nine samples. Additionally, Analysis of Similarities (ANOSIM) was applied to evaluate both inter-group and intra-group differences across the 9 samples. Pairwise comparisons between different treatment groups were performed using the DESeq2 R package (version 1.16.1). Genes with |log_2_(FoldChange)| > 1 and FDR < 0.05 were considered differentially expressed genes (DEGs).

### 2.6. Enrichment Analyses

To obtain functional annotations, the DEGs identified were mapped to the Gene Ontology (GO) and Kyoto Encyclopaedia of Genes and Genomes (KEGG) databases. GO term enrichment analysis was conducted to calculate the number of genes associated with each term, producing GO functional gene lists and gene count statistics. Additionally, KEGG pathway analysis was performed to further explore the biological functions of the DEGs in a pathway-based context.

### 2.7. STEM Analysis

Short Time-series Expression Miner (STEM) analysis [35] is a method used to cluster genes with similar expression trends across multiple continuous samples (minimum of three). Using STEM software and normalized expression data, DEGs exhibiting similar expression patterns were clustered, allowing the identification of gene sets that align with specific biological characteristics.

Protein–protein interaction (PPI) network analysis was performed for the differentially expressed genes (DEGs) within each significant expression profile using the STRING v10 database, aiming to identify modular networks and prioritize key genes. Subsequently, the top 200 genes were selected to construct gene co-expression networks, which were visualized using Cytoscape v3.10.1. Finally, potential hub genes were identified based on their highest connectivity with other genes within the network.

### 2.8. Quantitative Real-Time PCR (qRT-PCR) Validation of Transcriptomic Data

To validate the reliability of the RNA-seq results, ten differentially expressed genes (DEGs) were randomly selected for quantitative real-time PCR (qRT-PCR) analysis. The *RPL32* gene exhibited relatively low variation in Ct values and stable expression across samples; therefore, it was chosen as the internal reference gene for data normalization in this study. Specific primers were designed using Primer Express® Software v3.0.1 based on transcript sequences obtained from RNA-seq (Table 1).

Total RNA was extracted from the samples using the TRIzol reagent (Invitrogen, Carlsbad, CA, USA), and the RNA concentration and integrity were assessed using a NanoDrop micro-spectrophotometer and agarose gel electrophoresis, respectively. Reverse transcription was performed using the PrimeScript™ RT reagent Kit with gDNA Eraser (TaKaRa Bio, Kusatsu, Japan, #RR047A) according to the manufacturer’s instructions.

The qRT-PCR reaction was conducted in a 20 μL volume containing 8.2 μL of RNase-free water, 10 μL of Luna® Universal qPCR Master Mix (New England Biolabs, Ipswich, MA, USA, #M3003), 1 μL of cDNA template, and 0.4 μL each of the forward and reverse primers. Reactions were carried out on a CFX Connect™ Real-Time PCR Detection System (Bio-Rad Laboratories, Hercules, CA, USA) under the following cycling conditions: initial denaturation at 95 °C for 3 min, followed by 40 cycles of 95 °C for 10 s, 55 °C for 20 s, and 72 °C for 20 s, with a final extension at 75 °C for 5 s. Melt curve analysis was performed after each reaction to confirm the specificity of amplification. Each sample was analyzed in triplicate (biological replicates), and each qRT-PCR run included a no-template control (NTC). The relative gene expression levels were calculated using the 2^−ΔΔCT^ method based on well-defined amplification and melt curves.

The data were based on three biological replicates and three technical replicates. Statistical analysis was performed using GraphPad Prism v8.0.2 with an unpaired two-tailed *t*-test, and results were expressed as mean ± standard error of the mean (SEM) with statistical significance set at *p* < 0.05. In addition, expression trends from qPCR and RNA-Seq were visually compared to assess consistency. The consistency between qRT-PCR and RNA-seq results was evaluated to confirm the reliability of the transcriptomic data.

## 3. Results

### 3.1. Sequencing Data Statistics and Quality Analysis

Using Illumina RNA sequencing, a total of 466,211,548 raw reads were generated. After trimming adapter sequences and filtering out low-quality reads, 465,039,356 clean reads were retained. The Q20 and Q30 of each sample ranged from 97.46–98.19% and from 92.59–94.64%, respectively, with a GC content of 50.80–51.51% (Table 2). Following the removal of reads mapped to the rRNA database, 464,731,512 high-quality clean reads were obtained. These reads were subsequently aligned to the *T*. *ovatus* reference genome, yielding a total mapped and unique mapped mapping rate of 92.55–93.35% and 87.91–88.08% (Table 3). These results demonstrate that the sequencing, quality control, and alignment processes generated high-quality transcriptomic data, ensuring the reliability of downstream analyses.

**Figure 1:**
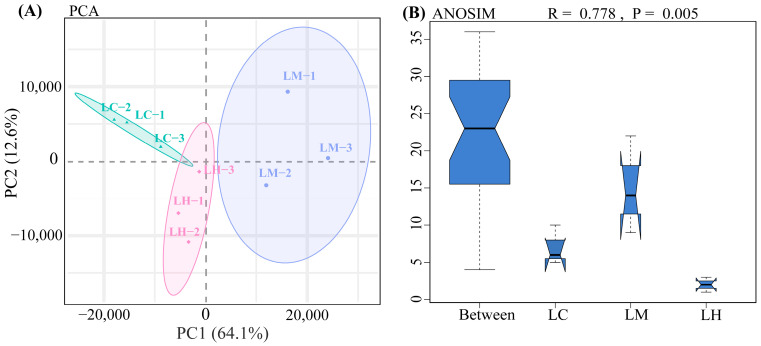
(**A**) PCA analysis results. Green, purple, and pink circles represent the confidence ellipses for the LC, LM, and LH groups, respectively. (**B**) ANOSIM analysis. The R value represents the ratio of between-group to within-group differences, with values closer to 1 indicating more pronounced differences between groups. The *p* value indicates the statistical significance of the difference, with *p* < 0.05 considered statistically significant. (For interpretation of the references to color in this figure legend, the reader is referred to the web version of this article).

PCA analysis revealed the tight clustering of samples within each group and clear separation between groups (Figure 1A). This pattern was further supported by ANOSIM analysis (R = 0.778, *p* = 0.005) (Figure 1B), indicating statistically significant differences between groups and minimal variation within groups. These results confirm the robustness and validity of the sample grouping.

### 3.2. Differential Expression Analysis

**Figure 2:**
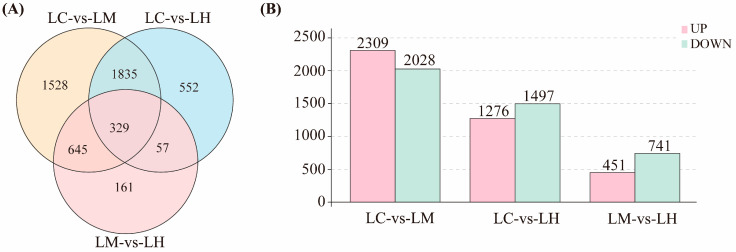
(**A**) Venn diagram showing the overlap of differentially expressed genes (DEGs) among three comparison groups: LC-vs-LM, LC-vs-LH, and LM-vs-LH; (**B**) Histogram showing the number of up-regulated and down-regulated DEGs in each comparison group.

Under varying flow speed stresses, a total of 5107 DEGs were identified across the three comparison groups (|log_2_(Fold Change)| > 1 and FDR < 0.05). Among these, 329 DEGs were common to all three comparison groups, 2537 DEGs were shared by two comparison groups, and 2241 DEGs were unique to individual groups (Figure 2A). The bar chart results reveal (Figure 2B) that 4337 DEGs were detected in the LC-vs-LM comparison (2309 up-regulated and 2028 down-regulated), 2773 DEGs were detected in the LC-vs-LH comparison (1276 up-regulated and 1497 down-regulated), and 1192 DEGs were detected in the LM-vs-LH comparison (451 up-regulated and 741 down-regulated). The highest number of DEGs was found in the LC-vs-LM comparison, suggesting that transcriptional responses between low and medium flow speeds are more pronounced. This may suggest that moderate water flow plays an important role in the response of *T*. *ovatus* to flow velocity stress. The differential gene expression levels for all comparison groups are available in the Supplementary Materials (Table S1).

### 3.3. GO and KEGG Enrichment Analyses

**Figure 3:**
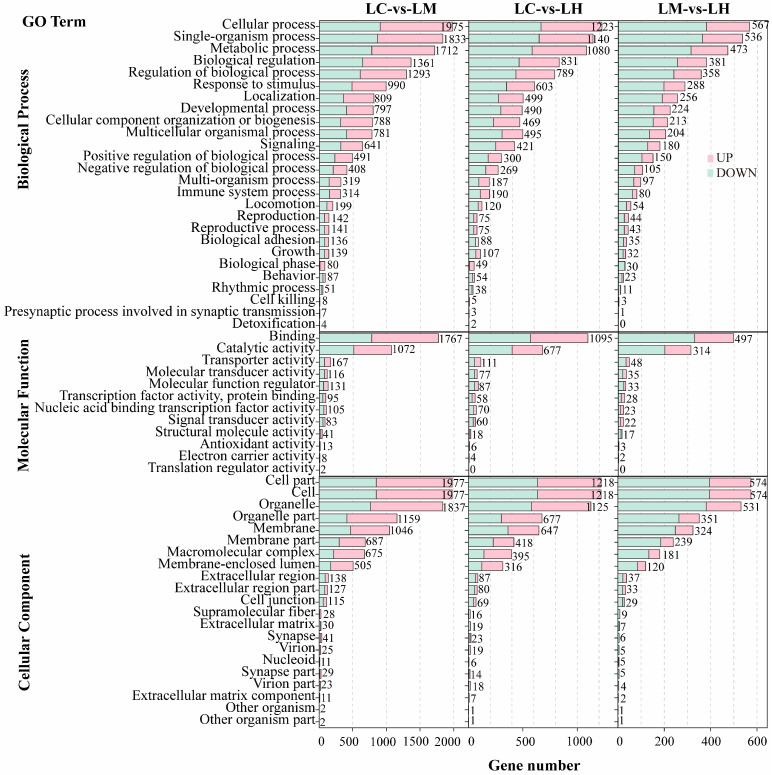
GO enrichment histograms of DEGs in LC-vs-LM, LC-vs-LH, and LM-vs-LH.

The GO enrichment analysis results were classified into three main categories: biological processes (BP), molecular functions (MF), and cellular components (CC). As shown in Figure 3, the top enriched GO terms in the BP category across all three comparison groups were cellular processes, single-organism processes, and metabolic processes. In the MF category, DEGs were mainly enriched in binding and catalytic activity. For the CC category, the most significantly represented terms were cell, cell part, and organelle.

**Figure 4:**
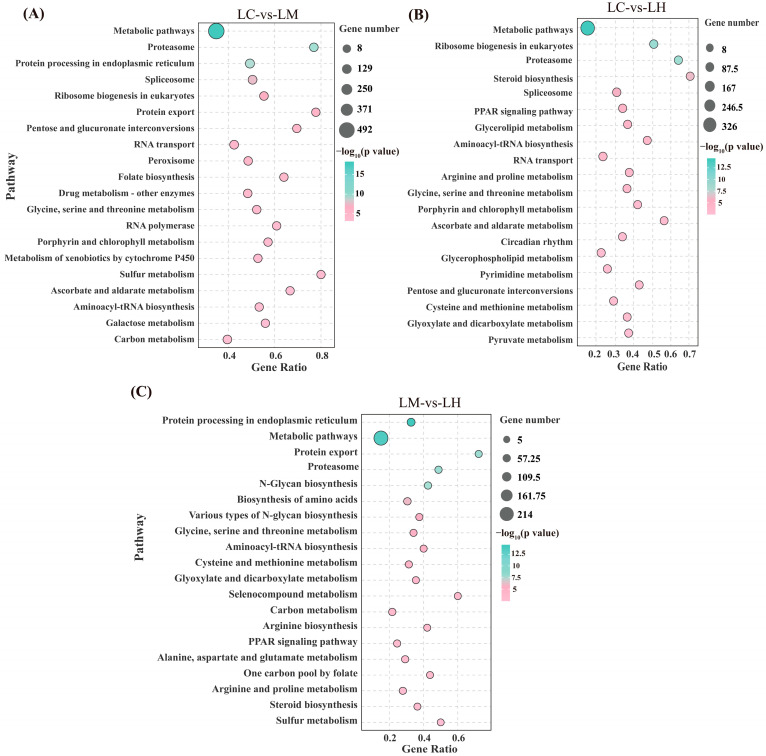
Bubble charts of the KEGG enrichment analysis of the top 20 pathways of the DEGs in the LC-vs-LM (**A**), LC-vs-LH (**B**), and LM-vs-LH (**C**).

The KEGG enrichment analysis results (Figure 4) revealed significant pathway enrichment across all comparison groups. In the LC-vs-LM group, 58 significantly enriched pathways (*p* < 0.05) were identified, primarily related to metabolic pathways, the proteasome, protein processing in the endoplasmic reticulum, the spliceosome, ribosome biogenesis in eukaryotes, protein export, and the peroxisome. In the LC-vs-LH group, 47 significantly enriched pathways (*p* < 0.05) were detected, mainly involving metabolic pathways, the proteasome, ribosome biogenesis in eukaryotes, steroid biosynthesis, the spliceosome, glycerolipid metabolism, and aminoacyl-tRNA biosynthesis. The LM-vs-LH comparison revealed 29 significantly enriched pathways (*p* < 0.05), including protein processing in the endoplasmic reticulum, the proteasome, protein export, N-glycan biosynthesis, metabolic pathways, and aminoacyl-tRNA biosynthesis. These results underscore the substantial involvement of pathways related to lipid and protein metabolism, indicating that flow velocity stress significantly affects these biological processes in *T*. *ovatus*.

### 3.4. Gene Expression Trend Profiles and Enrichment Analysis

**Figure 5:**
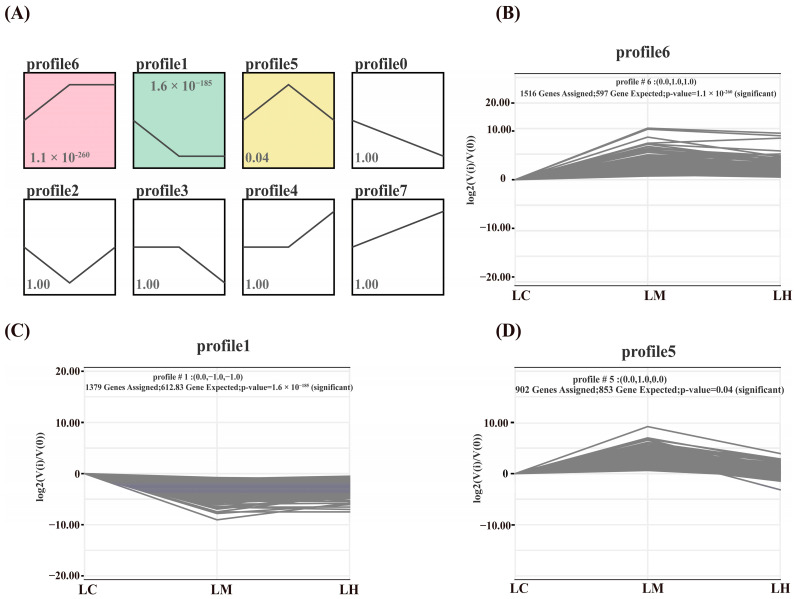
(**A**) Analysis of gene expression trend, (**B**) Expression trends for genes in Profile 6, (**C**) Expression trends for genes in Profile 1, (**D**) Expression trends for genes in Profile 5.

A total of three significantly enriched gene expression profiles (*p* < 0.05) were identified through STEM analysis: Profile 6 (*p* = 1.1 × 10^-260^), Profile 1 (*p* = 1.6 × 10^−185^), and Profile 5 (*p* = 0.04) (Figure 5). Among them, Profile 6 and Profile 1 exhibited completely opposite expression patterns. Profile 6 and Profile 1 contained 1516 and 1379 genes, respectively. Compared to the LC group, the majority of DEGs in Profile 6 were significantly up-regulated in both the LM and LH groups, whereas the genes in Profile 1 were markedly down-regulated in these two groups. Profile 5 included a total of 902 genes, with expression levels peaking in the LM group and remaining relatively low in the LC and LH groups.

**Figure 6:**
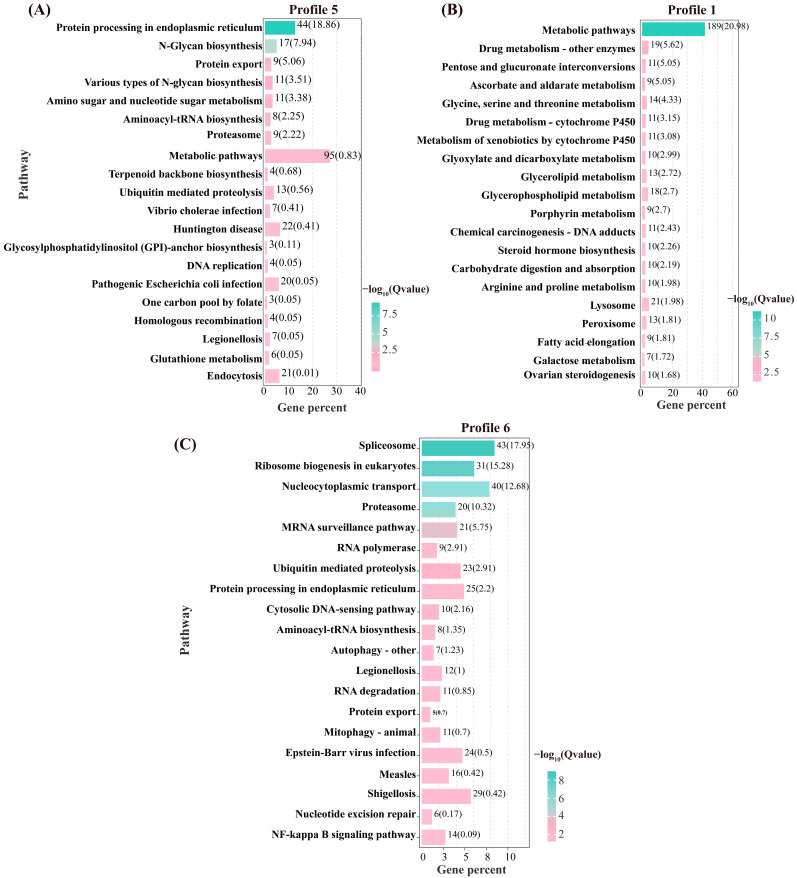
Top 20 pathways for KEGG enrichment analysis of DEGS in Profile 5 (**A**), Profile 1 (**B**), and Profile 6 (**C**).

To identify pathways significantly associated with flow velocity stress, KEGG enrichment analysis was performed on the genes in Profiles 6, 1, and 5 (Figure 6). In Profile 6, the significantly enriched pathways included spliceosome, ribosome biogenesis in eukaryotes, proteasome, ubiquitin-mediated proteolysis, and protein processing in the endoplasmic reticulum, among others. For Profile 1, the enriched pathways were mainly associated with glycine, serine, and threonine metabolism, glycerolipid metabolism, glycerophospholipid metabolism, steroid hormone biosynthesis, and pyruvate metabolism, among others. In Profile 5, the significantly enriched pathways included protein processing in the endoplasmic reticulum, N-Glycan biosynthesis, and aminoacyl-tRNA biosynthesis, among others.

### 3.5. PPI Analysis

**Figure 7:**
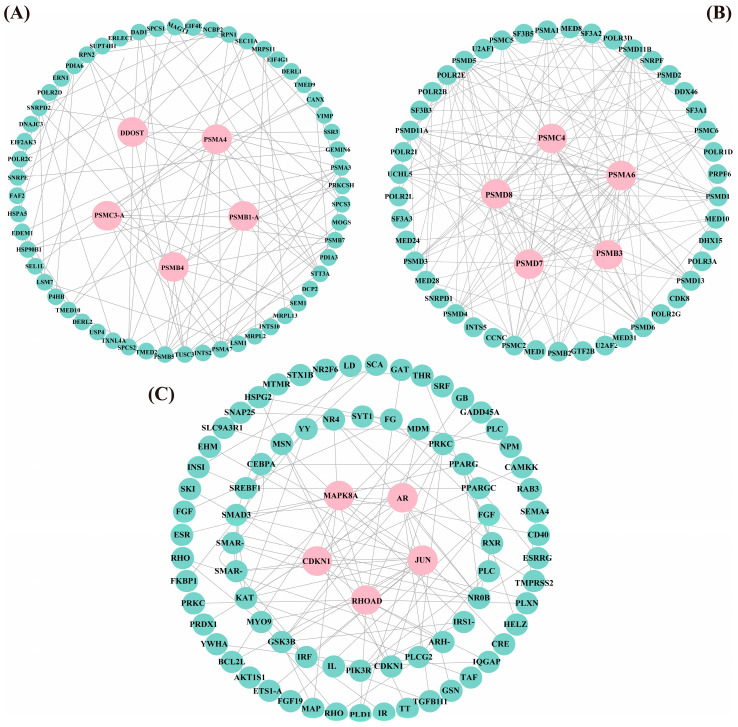
PPI network diagrams of the genes in Profile 5 (61 genes, 200 edges; (**A**)), Profile 6 (50 genes, 200 edges; (**B**)), and Profile 1 (80 genes, 200 edges; (**C**)). Hub genes are shown in pink, while other genes are shown in green. (For interpretation of the references to color in this figure legend, the reader is referred to the web version of this article).

Protein–protein interaction (PPI) network analysis identified distinct interaction networks within each expression profile (Figure 7). Profile 1 comprised 80 protein nodes, including five hub genes: *MAPK8A*, *RHOAD*, *AR*, *JUN*, and *CDKN1A*. Profile 5 contained 61 protein nodes with the hub genes *DDOST*, *PSMA4*, *PSMC3-A*, *PSMB4*, and *PSMB1-A*. Profile 6 encompassed 50 protein nodes, highlighting *PSMC4*, *PSMA6*, *PSMB3*, *PSMD7*, and *PSMD8* as central hub genes. These 15 hub genes exhibited strong interactions with other differentially expressed genes within their respective profiles, suggesting their critical roles in the transcriptional regulation of *T*. *ovatus* in response to flow velocity stress.

### 3.6. Validation of Gene Expression Patterns by qRT-PCR

**Figure 8:**
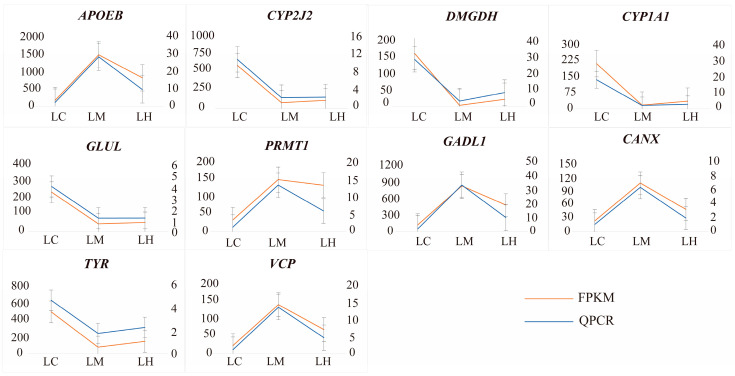
qRT-PCR validation results of 10 randomly selected genes. The right Y-axis represents the relative gene expression levels obtained by RT-qPCR using the 2^−ΔΔCT^ method, while the left Y-axis represents the relative expression levels from RNA-Seq data based on the FPKM method.

To verify the accuracy of the sequencing results, ten selected DEGs were analyzed using qRT-PCR. The line charts (Figure 8) demonstrate that the relative expression levels from qRT-PCR were consistent with those from the RNA-Seq data. Thus, these findings validate the reliability of our sequencing results.

## 4. Discussion

**Figure 9:**
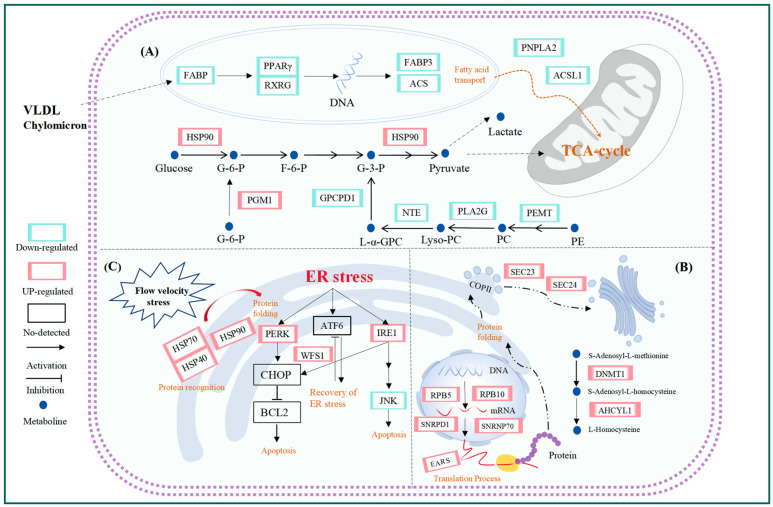
Some pathways affected by water velocity stress: (**A**) lipid metabolism; (**B**) protein metabolism; (**C**) Correct protein folding and UPR reactions.

To elucidate the molecular mechanisms underlying water flow induced stress, transcriptomic analyses were performed on the liver tissues of *T. ovatus* under static, moderate, and high water flow conditions. A total of 5107 DEGs were identified, along with three significant gene expression profiles and 15 hub genes obtained from PPI analysis. Integrated analysis revealed that the biological pathways closely associated with flow induced stress were mainly involved in glycolipid metabolism, protein synthesis, proteostasis, and endoplasmic reticulum (ER) stress (Figure 9). These changes were reflected in the altered expression levels of genes related to the PPAR signaling pathway, glycerolipid metabolism, glycerophospholipid metabolism, glycolysis, proteasome, spliceosome, aminoacyl-tRNA biosynthesis, and protein processing in the endoplasmic reticulum.

### 4.1. Effects of Flow Velocity Stress on Glucose and Lipid Metabolism

Water flow changes significantly affect the metabolic demands of fish [36]. Fish living in flowing waters exhibit a preference for certain flow conditions, allowing them to conserve energy by utilizing the water flow; however, in some cases, they may need to expend additional energy. Studies have shown that as flow velocity increases, fish must expend more energy to maintain swimming motion and balance [37]. In natural marine environments, fish often move toward areas with optimal flow conditions to minimize energy expenditure [38]. David Johansson et al. discovered that when salmon are exposed to strong water currents, they shift from their typical circular swimming patterns to upstream swimming, maintaining fixed positions, reflecting an energy optimization strategy in response to high flow environments [38]. In this experiment, it was observed that the tail-beat frequency of *T*. *ovatus* increased with the flow velocity and exhibited rheotaxis when the flow reached 90 cm/s. Previous studies have demonstrated that flow-induced rheotaxis significantly increases energy expenditure in fish [39]. Therefore, it is essential to investigate the effects of flow velocity stress on energy metabolism in *T. ovatus*. The liver, as a key metabolic organ in fish, plays a critical role in their adaptation to environmental changes by regulating energy supply and metabolite levels [29]. When fish are subjected to environmental stressors such as hypoxia, high temperatures, or salinity fluctuations, they balance energy demands by regulating glucose and lipid metabolism, thereby adapting to environmental changes and sustaining vital functions [40,41,42].

In this study, we identified two important signal pathways related to lipid metabolism: the PPAR signaling pathway and the glycerolipid metabolism pathway. The PPAR signaling pathway influences lipid metabolism by regulating genes involved in lipid synthesis, lipid decomposition, and lipid transport [43]. The expression levels of *FABP*, *PPARγ*, *RXRG*, *FABP3*, and *ACS* were down-regulated in the PPAR signaling pathway. *PPARγ* is a nuclear hormone receptor superfamily, and forms a heterodimer with the retinoid X receptor (RXR) encoded by the RXRG [44]. *PPARγ*/*RXR* complex directly regulates the transcription of lipid metabolism-related genes such as *FABP3* and *ACS* by binding to the peroxisome proliferator response element (PPRE) in the promoter region of target genes [45]. Therefore, the down-regulation of *PPARγ* and *RXRG* may inhibit the transcriptional activity of downstream genes like *FABP3* and *ACSL1*, thereby affecting lipid metabolism. In the glycerolipid metabolism pathway, the simultaneous down-regulation of *PNPLA2* and *ACSL1* further supports this mechanism. Triglycerides (TG) are the primary storage form of fatty acids in organisms and free fatty acids (FFA), which serve as the dominant substrates for fatty acid β-oxidation [46]. *PNPLA2* (Adipose Triglyceride Lipase, ATGL), the rate-limiting enzyme of lipolysis, specifically catalyzes the initial step of TG hydrolysis to generate diglycerides (DAG) and FFA [47]. The suppression of *PNPLA2* expression consequently diminishes FFA availability, thereby limiting the substrate supply for fatty acid β-oxidation. *ACSL1* (long-chain acyl-CoA synthetase 1) catalyzes the binding of long-chain fatty acids coenzyme A, forming activated acyl-CoA, which is a necessary step enabling fatty acids to enter the mitochondria for fatty acidv β-oxidation [48]. Therefore, the transcriptional suppression effect of the *PPARγ*/*RXR* complex, along with the down-regulation of *PNPLA2* and *ACSL1* genes, indicates that the lipid metabolism and energy production efficiency in *T*. *ovatus* are significantly reduced under flow velocity stress.

Exercise significantly enhances the utilization of carbohydrates in fish [49]. Glycolysis, as one of the key metabolic pathways in fish, rapidly breaks down glucose to produce ATP, providing energy for the organism [50]. Our transcriptomic sequencing results showed that glycolysis-related genes were significantly upregulated in the liver of *T*. *ovatus* (peak at medium flow velocity). The *GCK* gene encodes glucokinase, the rate-limiting enzyme of the glycolytic pathway, which catalyzes the phosphorylation of glucose to produce glucose-6-phosphate (G6P) [51]. The *PGM1* encodes phosphoglucomutase 1 (PGM1), which catalyzes the bidirectional conversion between glucose 1-phosphate (G1P) and G6P [52]. The *PDHA1* encodes the pyruvate dehydrogenase complex, responsible for converting pyruvate produced by glycolysis into acetyl-CoA, which then enters the TCA cycle to continue generating ATP [53]. Therefore, the up-regulation of these genes suggests that glycolysis plays an important role in the energy supply of *T*. *ovatus* in response to changes in flow velocity. Previous studies have reported that during exhaustive exercise, anaerobic metabolism is significantly enhanced in fish and shrimp, while aerobic metabolic activity is inhibited [54,55]. Lactate production has long been considered an important indicator of anaerobic metabolic capacity. In our experiment, we found that as the water flow velocity increased, the glucose levels in the liver of *T*. *ovatus* showed no significant change, but lactate levels increased [14]. Similarly, in a study by Tingyao Zhu et al., it was found that the metabolic mode of mandarin fish (*Siniperca chuatsi*) gradually shifted from aerobic metabolism to anaerobic metabolism under acute flow velocity stress, with the activation of the anaerobic glycolytic pathway to provide energy for the organism [37]. Based on these findings, we speculate that when *T*. *ovatus* faces increased water flow, its energy metabolism gradually shifts from aerobic to anaerobic, adapting to the energy demands of movement by activating the glycolytic pathway. However, in the study by Shuo Li et al., the expression of key genes involved in glucose and lipid metabolism (such as *GCK* and *ANGPTL4*) was downregulated in spotted sea bass (*Lateolabrax maculatus*) under high-flow-velocity conditions, suggesting the possible prioritization of immediate metabolic adjustments and stress response mechanisms [56]. In contrast to our findings, this discrepancy indicates that the regulatory mechanisms of energy metabolism in response to flow stress may vary across species or flow intensities, reflecting distinct adaptive strategies in different fish.

Additionally, our transcriptomic data show a down-regulation in the expression of *PEMT*. *PEMT* (Phosphatidylethanolamine N-methyltransferase) is a key enzyme that catalyzes the methylation of phosphatidylethanolamine (PE) to produce phosphatidylcholine (PC) through three methylation reactions [57]. PC is the most abundant phospholipid in mitochondria, and research has confirmed that PC plays a crucial role in maintaining cell membrane fluidity [58]. The inhibition of *PEMT* expression directly leads to a reduction in PC biosynthesis, thereby decreasing the flexibility and adaptability of cell membranes. Cell kinetics studies have demonstrated that cells cope with environmental stress by modifying self-mechanical properties. Specifically, when variable environment conditions are changed, cells decrease their volume and increase their hardness [59]. The above suggests that, when subjected to medium and high water velocity stress, *T*. *ovatus* might change the elasticity of its cell membranes and harden these to actively respond to water velocity stress and minimize its impact. Thus, under moderate and high flow velocity stress, *T*. *ovatus* ovatus may alter the elasticity of its cell membranes, making them stiffer as an active response to water velocity pressure to mitigate its impact. Furthermore, *PLA2G16*, *NTE*, and *GPCPD1* were also found to have a down-regulated expression. *PLA2G16* and *NTE* participate in catalyzing the hydrolysis of PC, providing precursors for subsequent lipid synthesis reactions. *GPCPD1*, which encodes glycerol-3-phosphate dehydrogenase 1, catalyzes the production of glycerol phosphate from glycerol-3-phosphate, a critical process in the synthesis of phospholipids and glycerol [60]. Based on these findings, we speculate that *T*. *ovatus* reduces the synthesis of PC and glycerophosphates, thereby lowering cell membrane fluidity and increasing membrane hardness, which enhances its adaptability to water velocity stress. This is an adaptive response of *T*. *ovatus* to flow velocity stress.

### 4.2. Effects of Flow Velocity Stress on Protein Synthesis

Protein synthesis mainly involves transcription, translation, modification, and processing. At the protein transcription stage, there are three main types of RNA polymerases, which are responsible for transcribing all the RNA needed in eukaryotic cells [61]. Among them, RNA polymerase I is mainly responsible for the processing and synthesis of ribosomal RNA (rRNA). HnRNA, synthesized by RNA polymerase II, is the precursor of messenger RNA (mRNA), and RNA polymerase III can catalyze the production of transfer RNA (tRNA) and microRNA (miRNA).

At the transcription initiation stage, the expression of shared subunits of RNA polymerases I, II, and III-related genes (*RPB5*, *RPB10*), the core subunit of RNA polymerase I-related gene (*RPC19*), the core subunit of RNA polymerase II-related genes (*RPB2*, *RPB7* and *RPB9*), and the core subunit of RNA polymerase III-related genes (*RPC19*, *RPC1*, *RPC4* and *RPC11*) were significantly up-regulated in the LM and LH groups. In addition, the transcription factors of RNA polymerase II include the TFIIB, TFIIE, TFIID and TFIIH families. The *TAF5* gene, which encodes transcription factors TFIIB and TFIID, as well as the *TFIIE1/TFIIE2* genes, which encode the TFIIE family of transcription factors, and the *CDK7* gene, which encodes the TFIIH family of transcription factors, were also up-regulated. Transcription factors are proteins that regulate the rate of transcription by binding to DNA regulatory sequences [62]. The up-regulation of all of the above genes implies an accelerated rate of transcription.

At the stage of processing transcription products (such as mRNA, rRNA, and tRNA), the mRNA is removed from the intronic portion, making the exon a continuous sequence by variable shearing [63]. The spliceosome generally consists of small nuclear RNAs (U1, U2, U4, U5, and U6) that collectively form an RNA–protein complex. Compared to the LC group, 43 genes encoding nuclear small RNA (such as *SNRPD1*, *SNRNP70*, *LSM4*, and *PRPF6*) were up-regulated in the LM and LH groups. Spliceosomes play a constant role in this process. Moreover, the processed products (such as mRNA, rRNA, tRNA) are transported from the nuclear pore to the cytoplasm for translation via the RNA transport pathway. At this time, 40 genes (such as *PRMT5*, *EIF5B,* and *KPNB1*) in the nuclear pore complex (NPC), the motor neuron survival complex (SMN complex), translation initiation factor (EIFs), exon junction (EJC), and transcription export complex (TREX), which are related to transport biological processes, were also up-regulated. This indicated that transport activity was enhanced.

At the translation stage, mRNAs are templates for protein synthesis, while tRNAs are tools for amino acid translocation. Compared to the LC group, some genes (such as *EARS*, *VARS*, *YARS*, *WARS*, and *CARS2*) encoding various aminoacyl-tRNA synthetases were up-regulated in the LM and LH groups. Different aminoacyl-tRNA synthetases play important roles in helping tRNAs recognize substrate amino acids and their corresponding tRNAs. The mRNA surveillance pathway, on the other hand, has a role in detecting and degrading aberrant mRNAs [64]. Here, nonsense-mediated mRNA decay (NMD) is an important post-transcriptional gene expression regulatory mechanism in eukaryotic cells, which can control cellular tissue homeostasis by detecting substandard mRNAs and triggering degradation, thereby preventing the accumulation of truncated proteins [65,66]. Genes related to NMD (such as *RNMT*, *PABPN1*, *ETF3*, *SMG1* and *SMG5*) were also up-regulated, indicating that the stable synthesis of high-quality proteins were maintained by constantly eliminating unqualified mRNA.

In the modified processing stage, the newly generated peptide chain enters the endoplasmic reticulum (ER) through the translocation channel by co-translational translocation before undergoing further processing. Subsequently, folded proteins are packaged into transport coat protein complex II (COPII), which shuttles them to the Golgi complex [67]. The COPII consists of the small-molecule GTPase SAR1, Sec23/24 complex, and Sec13/31 complex [68], which are initiated after the activation of small GTPase SAR1. Compared to the LC group, the *SEC61A* and *SEC63* genes encoding post-translational translocation channels, the *RRBP1* gene encoding the ribosome-binding protein p180 in endoplasmic reticulum (ER) membrane [69], the *SEC23* and *SEC24* genes encoding the COPII, and the *SEC12* gene encoding the small-molecule GTPase SAR1 were up-regulated in the LM and LH groups. However, the misfolded proteins are retained within the ER lumen for ER-associated degradation (ERAD). Subsequently, the misfolded proteins are transported back to the cytoplasm through the dislocon channel and degraded by the proteasome [70]. Among them, the glycoprotein glucosyltransferase encoded by the *UGGT1* gene can prevent misfolded proteins from being transported out of the ER, which is a key enzyme in ER quality control [71]. ERAD-related genes (such as *DNAJA1*, *DNAJA2*, *UBX1*, *VCP*, *NPLOC4*, *HSPBP1*, *UBQLN*, *RAD23*), and genes encoding proteasomes (such as *PSMA1*, *PSMA6*, and *PSMB2*), were also up-regulated. The above reactions indicated that *T*. *ovatus* responded to water velocity stress by enhancing protein synthesis.

The methionine cycle is an important process by which organisms provide methyl for extensive methylation reactions. Furthermore, methylation is an important means of post-translational modification in proteins, which can control protein stability. Compared to the LC group, DNA methyltransferase 1 and adenosylhomocysteinase encoded by the *DNMT1* and *AHCYL1* genes, respectively, which are involved in the methionine cycle and provide methyl for widespread methylation reactions in the body (such as protein synthesis), were up-regulated in the LM and LH groups. DNA methyltransferase 1, a DNA methylation protein, is primarily responsible for maintaining methylation [72]. As a universal biological factor, S-adenosyl-L-methionine (SAM) can transfer methyl to proteins, nucleic acids, and lipids [73]. Methylation typically occurs on amino acid residues such as lysine and arginine as part of the post-translational modification of proteins [74]. There are nine known protein arginine methyltransferases (PRMTs), including *PRMT1-9* [75]. The expression level of the *PRMT1*, *PRMT4,* and *PRMT5* genes were up-regulated, suggesting that the methylation activity of arginine was enhanced. The methylation of arginine plays a key role in mRNA translation, pre-mRNA splicing, and cellular signal transduction [76], which correlates with the protein processing described above, once again suggesting that *T*. *ovatus* responded to water velocity stress by enhancing protein synthesis.

### 4.3. Protein Folding and Unfolded Protein Response (UPR)

In the endoplasmic reticulum (ER), molecular chaperones and folding enzymes assist newly synthesized proteins in folding correctly, which is essential for the proper functioning of cells [77]. Heat shock proteins (HSPs) are a class of molecular chaperones, including HSP110, HSP90, HSP40, HSP70, and sHSP. When cells are subjected to environmental stressors such as heat, hypoxia, and ultraviolet radiation, the expression of HSPs increases, assisting other proteins in proper folding, assembly, and degradation, and preventing the aggregation of misfolded proteins, thereby maintaining protein homeostasis [78]. Thus, HSPs are crucial for cell protection. They are induced during cellular stress and participate in multiple important biological processes such as protein folding and processing, apoptosis, and receptor-mediated signal transduction. Through these regulatory mechanisms, HSPs help the organism withstand adverse environmental conditions and protect cells from damage [79].

This study found that under flow velocity stress, the expression of HSP-encoding gene families in the liver of *T*. *ovatus*, including HSP70 (*HSPA5*), HSP90 (*GRP94*), and HSP40 (*DNAJA1*, *DNAJA2*, *DNAJB12*, *DNAJC3*, *DNAJC5*), was significantly up-regulated (peak at medium flow velocity) in both the moderate and high flow velocity groups. HSPA5, also known as Heat Shock Protein Family A Member 5, is typically expressed at low levels under normal conditions, but its expression increases significantly during stress [80]. When misfolded or unfolded proteins accumulate, *HSPA5* assists in their degradation and, through interaction with HSP40 family members, promotes correct protein folding [81]. *HSP40* functions as a co-chaperone for HSPA5, aiding its dissociation from protein substrates and allowing HSPA5 to continue its molecular chaperone activity [82]. This cooperative mechanism ensures efficient protein quality control and helps maintain cellular proteostasis. The HSP90 family comprises four subtypes: HSP90α and HSP90β, localized in the cytoplasm; Trap1, localized in mitochondria; and GRP94, localized in the endoplasmic reticulum. GRP94 plays a key role in protein folding, stress signal transduction, and the activation of the unfolded protein response (UPR) in the endoplasmic reticulum [83]. The up-regulation of HSP genes observed under flow velocity stress likely supports the maintenance of protein homeostasis in *T. ovatus*, protecting cells from damage. In a study by K. Anttila, it was found that heat stress significantly increased the expression levels of HSP genes (*HSP90α* and *HSP70*) in the heart of sockeye salmon, and similar increases were observed under swimming stress conditions [84].

ER is highly sensitive to external stimuli, and when these stressors impair its protein-folding function, unfolded or misfolded proteins accumulate in the ER lumen, triggering the unfolded protein response (UPR) to prevent cellular toxicity [85]. This response involves three UPR sensors: IRE1α, PERK, and ATF6α, which work together to reduce the protein-folding load while enhancing the cell’s capacity to manage ER stress. However, when ER stress becomes excessive or prolonged, these pathways shift from promoting cell survival to triggering cell death, ultimately leading to ER-induced apoptosis [86]. In a study by Zhao et al., heat stress was found to induce hepatocyte apoptosis in Micropterus salmoides through the IRE1α/TRAF2/ASK1/JNK pathway. The sustained activation of this pathway indicated that heat stress (HS) significantly triggered ER stress (ERS), which eventually led to apoptosis [87]. In the present study, UPR-related genes such as *PERK*, *WFS1*, and *IRE1* were significantly up-regulated under moderate and high-flow-velocity conditions, indicating that flow velocity stress activated the UPR, allowing cells to cope with ER stress by restoring protein homeostasis. Notably, when the flow velocity further increased to 90 cm/s, the expression of these genes began to decline, while the anti-apoptotic factor *JNK* was also significantly down-regulated. This phenomenon suggests that the adaptive response of the unfolded protein response (UPR) in the endoplasmic reticulum of *T*. *ovatus* may weaken under high flow velocities (≥90 cm/s), indicating that the cells’ ability to cope with flow velocity stress may be reaching its limit.

In our previous study on the effects of flow velocity on the swimming behavior and exercise physiology of *T*. *ovatus*, we found that the critical swimming speed of *T*. *ovatus* is 99.78 ± 12.66 cm/s [14]. When the flow velocity approaches or exceeds the critical swimming speed, the fish must mobilize large amounts of energy to maintain swimming, which can lead to a sharp increase in metabolic load and an increase in the protein-folding burden on the endoplasmic reticulum. If the flow velocity continues to rise, the fish’s cells may be unable to continuously and effectively address the protein-folding issues in the endoplasmic reticulum, causing the UPR response to gradually weaken and thereby triggering an ER stress response. Therefore, we hypothesize that when the flow velocity exceeds 90 cm/s, *T*. *ovatus* may struggle to maintain effective stress adaptation, resulting in a shift toward pro-apoptotic responses and initiating the apoptosis program.

In summary, under moderate to high-flow-velocity stress conditions, *T*. *ovatus* enhances its protein-folding capacity and ability to cope with endoplasmic reticulum (ER) stress by upregulating various HSPs and UPR-related genes. This response helps alleviate ER stress, maintain cellular homeostasis, and supports the fish’s ability to adapt to environmental changes. However, when the flow velocity increases to 90 cm/s, the expression levels of UPR-related genes decrease, and the anti-apoptotic factor *JNK* is also significantly down-regulated, indicating that the cellular adaptive response has reached its limit and can no longer effectively cope with higher flow velocity stress. This change suggests that beyond this flow velocity threshold, cells may be unable to maintain normal protein folding and energy metabolism, leading to exacerbated ER stress and potentially triggering apoptotic signaling. The molecular regulatory mechanism by which the UPR shifts from a protective response to apoptosis is a complex biological process involving the coordination and transition of multiple signaling pathways. How *T*. *ovatus* finely regulates this mechanism to adapt to flow velocity stress warrants further investigation.

## 5. Conclusions

This study shows that flow velocity stress significantly affects key biological processes in *T*. *ovatus*, such as glucose and lipid metabolism, protein synthesis, protein homeostasis, and the endoplasmic reticulum stress response. Under flow velocity stress, *T*. *ovatus* adjusts the expression of genes related to lipid metabolism and glycolysis to fuel swimming, while maintaining metabolic homeostasis and membrane stability. The enhancement of protein synthesis improves the efficiency of biochemical reactions involved in energy metabolism, which is crucial for the species’ adaptation and sustained performance under flow velocity stress. By upregulating HSPs and activating the Unfolded Protein Response (UPR), *T*. *ovatus* maintains protein homeostasis and prevents apoptosis, highlighting a protective strategy in response to stress.

Moreover, we confirmed that when the flow velocity reaches 90 cm/s, the expression of UPR-related genes weakens and anti-apoptotic genes are suppressed, indicating that the protective stress response of *T*. *ovatus* is negatively impacted. Prolonged exposure to this or higher velocities could disrupt homeostasis, potentially leading to apoptosis and irreversible liver damage. Therefore, in practical farming, deep-sea cages should be set in areas where the current velocity is below 90 cm/s to avoid long-term negative effects on the physiological functions of the fish.

## Figures and Tables

**Table 1:** Primer sequences of selected genes for qRT-PCR.

Gene Name	Forward Primer (5′-3′)	Reverse Primer (5′-3′)	Amplicon Size (bp)
*RPL32*	ACCAAGTACATGCTGCCAAC	CCTGTTCTTGGAGGAGACGT	129
*APOEB*	AGCTCTGATCCTTGCTCTGG	CTGCCAGAAACGATCCACAG	109
*CYP2J2*	GACTCAACCTGCCCTACACT	ATGAAGTAACCACCCAGCGT	118
*DMGDH*	AAGGAGCTGTTTGAGTCGGA	TGGTCCAGACACGATGTTGA	111
*CYP1A1*	AGCAAACCTTACCTGAGCCT	CCTGCAAACTCATCCCCTTG	146
*GLUL*	AGAAGTGATGCCTGCTCAGT	AGGGATTGGTTTGGGGTCAA	148
*PRMT1*	GGTGACCATCATCAAGGGGA	GTTTCAGCCACTTGTCCCTG	146
*GADL1*	TGTACATGGTGTCGGCTGAT	TCCAAGCACTGTAGTCCCTG	131
*CANX*	AAAGCCAAACATCACGCCAT	CAGTTTGACGTAGGCACCAC	129
*TYR*	GCCACTCAAGGAACAGCTTC	AGTGGGGAGGTGAGACATG	131
*VCP*	GACCAGACATCATCGACCCT	CTTGCTGATGGGACTCTTGC	131

**Table 2:** Sequencing results of each sample.

Sample	Raw Reads	Raw Data (bp)	Clean Reads	Clean Data (bp)	Q20 (%)	Q30 (%)	GC (%)
LC-1	52,626,702	7,894,005,300	52,508,094	7,843,274,809	97.78	93.31	51.46
LC-2	53,981,850	8,097,277,500	53,860,000	8,046,760,233	97.75	93.26	51.08
LC-3	50,412,392	7,561,858,800	50,294,396	7,512,580,622	97.76	93.26	51.21
LM-1	50,056,706	7,508,505,900	49,939,602	7,458,492,045	97.85	93.49	50.80
LM-2	45,741,048	6,861,157,200	45,626,710	6,816,994,220	97.65	93.04	51.16
LM-3	48,930,144	7,339,521,600	48,814,618	7,290,965,392	97.79	93.34	51.08
LH-1	59,278,412	8,891,761,800	59,081,540	8,841,451,505	98.19	94.64	51.51
LH-2	53,104,482	7,965,672,300	52,962,484	7,914,830,626	97.46	92.59	51.05
LH-3	52,079,812	7,811,971,800	51,951,912	7,766,147,368	97.75	93.19	51.32

**Table 3:** Results of comparison between sample and reference genome.

Sample	High Quality Clean Reads	Unmapped (%)	Unique_Mapped (%)	Multiple_Mapped (%)	Total_Mapped (%)
LC-1	52,479,326	3,676,310 (7.01%)	46,224,285 (88.08%)	2,578,731 (4.91%)	48,803,016 (92.99%)
LC-2	53,819,202	3,614,444 (6.72%)	47,599,680 (88.44%)	2,605,078 (4.84%)	50,204,758 (93.28%)
LC-3	50,262,718	3,448,641 (6.86%)	44,418,126 (88.37%)	2,395,951 (4.77%)	46,814,077 (93.14%)
LM-1	49,902,958	3,568,531 (7.15%)	44,238,011 (88.65%)	2,096,416 (4.20%)	46,334,427 (92.85%)
LM-2	45,597,328	3,339,138 (7.32%)	40,250,550 (88.27%)	2,007,640 (4.40%)	42,258,190 (92.68%)
LM-3	48,784,062	3,632,330 (7.45%)	42,888,098 (87.91%)	2,263,634 (4.64%)	45,151,732 (92.55%)
LH-1	59,043,668	4,049,939 (6.86%)	52,098,221 (88.24%)	2,895,508 (4.90%)	54,993,729 (93.14%)
LH-2	52,923,028	3,708,476 (7.01%)	46,991,375 (88.79%)	2,223,177 (4.20%)	49,214,552 (92.99%)
LH-3	51,919,222	3,451,625 (6.65%)	46,075,841 (88.75%)	2,391,756 (4.61%)	48,467,597 (93.35%)

## Data Availability

The raw Illumina sequencing reads and transcript sequences have been uploaded to the NCBI SRA database under the accession number PRJNA1277463.

## Supplementary Materials

The following supporting information can be downloaded at: https://www.mdpi.com/article/10.3390/ani15131932/s1, Table S1: Differential gene expression levels among the comparison groups.
